# Engagement in Chemsex among Men Who Have Sex with Men (MSM) in Malaysia: Prevalence and Associated Factors from an Online National Survey

**DOI:** 10.3390/ijerph20010294

**Published:** 2022-12-24

**Authors:** Francesca Maviglia, Jeffrey A. Wickersham, Iskandar Azwa, Nicholas Copenhaver, Olivia Kennedy, Monique Kern, Antoine Khati, Sin How Lim, Kamal Gautam, Roman Shrestha

**Affiliations:** 1 Yale School of Medicine, Department of Internal Medicine, Section of Infectious Diseases, 135 College St., Suite 323, New Haven, CT 06510, USA; 2 Centre of Excellence for Research in AIDS (CERiA), Faculty of Medicine, University of Malaya, Kuala Lumpur 59100, Malaysia; 3 Infectious Diseases Unit, Department of Medicine, Faculty of Medicine, University of Malaya, Kuala Lumpur 59100, Malaysia; 4 Department of Allied Health Sciences, University of Connecticut, Storrs, CT 06269, USA; 5 Department of Social & Preventive Medicine, Faculty of Medicine, University of Malaya, Kuala Lumpur 59100, Malaysia

**Keywords:** chemsex, sexualized drug use, party and play, men who have sex with men, Malaysia, HIV prevention

## Abstract

Background: Chemsex is the use of illicit drugs—particularly methamphetamine, gamma hydroxybutyrate (GHB), and gamma-butyrolactone (GBL)—to enhance sexual activity. Chemsex, which occurs primarily among communities of men who have sex with men (MSM), is associated with greater HIV risk behaviors, including multiple sex partners, group sex, condomless sex, and injection drug use. Despite evidence showing chemsex engagement among Malaysian MSM, there is a paucity of research on chemsex among MSM in Malaysia. Methods: This cross-sectional study was conducted through an online survey (August to September 2021) among 870 Malaysian MSM. Participants were recruited through targeted advertisements on social networks. We collected information regarding participants’ recent (<6 months) engagement in chemsex, demographic characteristics, psychosocial factors, pre-exposure prophylaxis (PrEP) knowledge and history, and recent sexual- and drug-related behavior. Multivariable logistic regression was used to identify factors associated with recent (<6 months) chemsex engagement. Results: Just under 1 in 10 (9.0%) of participants reported having engaged in chemsex in the previous six months. More than two-thirds of participants (69.1%) had not disclosed their sexual orientation to anyone in their family and 35.2% reported moderate to severe depressive symptoms. Multivariable analysis found that recent injection drug use (adjusted odds ratio: aOR = 6.61; 95% confidence interval: CI, 2.30–19.03), having shared pre-exposure prophylaxis (PrEP) with someone else (aOR = 5.60; 95% CI, 1.76–17.77), higher perceived HIV risk (aOR = 3.15; 95% CI, 1.25–7.93), knowing someone using PrEP (aOR = 2.93; 95% CI, 1.62–5.31), recent engagement in transactional sex (aOR = 2.38; 95% CI, 1.06–5.39), having a recent STI diagnosis (aOR = 2.36; 95% CI, 1.25–4.44), recent engagement in anal sex (aOR = 2.21; 95% CI, 1.07–4.57), and recent sexual intercourse with an HIV-positive partner (aOR = 2.09; 95% CI, 1.07–4.08) were associated with recent engagement in chemsex. Conclusions: Malaysian MSM who practice chemsex are vulnerable to several HIV risk factors, such as transactional sex, HIV-positive sexual partners, and injection drug use. There is an urgent need for programs that integrate drug, sexual health, and mental health services, with a focus on harm reduction (e.g., condoms, access to and utilization of HIV testing and PrEP services, drug knowledge, and safer drug use) tailored for MSM who practice chemsex.

## 1. Introduction

Sexualized drug use (SDU) refers to the intentional use of illicit drugs for the purpose of enhancing a sexual experience. Although SDU occurs across genders and sexual orientations [1], chemsex (also referred to as ‘*Party and Play*’) is a particular SDU subculture among men who have sex with men (MSM). It is characterized by the use of a distinct set of drugs—typically, methamphetamine, mephedrone, gamma hydroxybutyrate (GHB), and gamma-butyrolactone (GBL) [1,2]—before or during sexual activities to facilitate, enhance, and prolong them [3,4]. Prevalence of recent chemsex among MSM varies across geographic and recruitment settings [5,6], ranging from 3% engagement in the past month in a study recruiting MSM from Scotland, Wales, Northern Ireland, and the Republic of Ireland [7], to 29% in the past 6 months among users of an MSM dating app in Amsterdam [8]. In recent years, chemsex among MSM has coincided with the emergence of digital geosocial technologies (e.g., hookup apps), creating new opportunities for finding chemsex partners [5,9,10].

Chemsex is a growing public health concern globally because of a higher risk of associated adverse health outcomes [2,10]. Existing literature further reports that chemsex users experience adverse health outcomes associated with the use of drugs, including biopsychosocial risks such as craving and withdrawal symptoms, overdose, dehydration, heart failure, nonconsensual acts, depression, anxiety, psychosis, and loss of job [3,11,12,13,14,15]. Additionally, engagement in chemsex has been associated with high-risk sexual behaviors, including the ability to have prolonged sexual sessions, having multiple sex partners, and engaging in condomless anal sex [3,5,16], resulting in greater vulnerability to sexually transmitted infections and HIV transmission, as well as other bloodborne infections (e.g., hepatitis C virus; HCV) [2,17]. In various settings, these direct harms are further compounded by criminalizing and stigmatizing same-sex sexual orientation and drug use, deterring chemsex users from fully accessing or utilizing health care services [18,19].

Notwithstanding these studies, there is a paucity of research on chemsex among MSM in Malaysia. This may be attributed to stigmatization and legal concerns around same-sex sexual behavior and drug use [20] that create barriers for MSM in Malaysia to participate in chemsex-related research or access resources provided by healthcare and lesbian, gay, bisexual, and transgender (LGBT) friendly community-based organizations. Malaysia is a transit country for drug trafficking, and the recent increase in illicit drug (e.g., amphetamine-type stimulants) seizures by law enforcement suggests the widespread availability of psychoactive substances [21,22]. To date, only one published study qualitatively explored the motivations for, and management of methamphetamine use among MSM from the Greater Kuala Lumpur region in Malaysia [19]. Considering this gap, we aimed to assess the prevalence of recent chemsex engagement among MSM and explore the drivers associated with chemsex involvement. The findings from this study are crucial to further characterize the subgroup of MSM chemsex users, better understand chemsex as a practice in this subpopulation and inform the development of tailored harm-reduction programs.

## 2. Methods

### 2.1. Study Design and Participants

A cross-sectional online survey assessing chemsex practices among Malaysian MSM was conducted from August to September 2021. The eligibility criteria for the study included: (i) being 18 years or older; (ii) self-reported HIV negative or HIV status unknown; (iii) being a cisgender male who has sex with men; and (iv) being able to read and understand English.

### 2.2. Study Procedures

MSM were recruited through convenience sampling via advertisements on the geosocial networking (GSN) app for gay men (i.e., Hornet) and a popular social networking website for the general population (i.e., Facebook). On the Hornet app, the advertisement was pushed as a message to the chat inboxes of all users residing in Malaysia. On Facebook, we posted flyers on the Facebook pages of non-governmental organizations (NGOs) and community-based organizations (CBOs) that provide services to MSM. These targeted advertisements appeared as either a static ad on the right-hand pane of the website or an ad that resembled a standard post in a user’s social media feed. Interested users who clicked on ads were directed to an eligibility self-screening tool and a brief online consent form hosted by Qualtrics (Qualtrics, Provo, UT, USA).

Each eligible participant completed an online consent form by acknowledging that they understood the purpose, risks, and benefits of the study prior to completing the survey. On average, participants took 35 min to complete the online survey. The study protocol and the consent form were approved by the University of Malaya Research Ethics Committee (UMREC) and the University of Connecticut Institutional Review Board.

During the 1-month recruitment period, a total of 1976 individuals opened the survey. Of those, 1799 (91.0%) met the inclusion criteria and consented to participate in the survey. Of the 1799 who started the survey, 929 (51.6%) responses were excluded due to incomplete responses and missing data, thus leaving the final analytic sample to 870 (48.4%). 

## 3. Measures

The primary outcome variable, recent engagement in chemsex, was defined as any use of ecstasy, crystal methamphetamine, ice, GHB/GBL, or foxy before or during sexual activity in the last 6 months.

Independent variables included participants’ demographic characteristics, psychosocial factors, pre-exposure prophylaxis (PrEP) knowledge and history, and recent (defined as within the previous 6 months) sexual behavior and other HIV-related behaviors.

### 3.1. Demographic Characteristics

We collected participants’ demographic characteristics such as age, ethnicity, educational status, relationship status, and income. Continuous variables such as age and income were recoded into categorical variables. Participants were assigned to one of four age groups (18–29; 30–39; 40–49; 50+), chosen to reflect the distribution by the age of new HIV infections in Malaysia, which are primarily concentrated among people aged 20–29 and 30–39 [23], to be able to explore whether the concentration of chemsex among particular age groups might have implications for the growing HIV epidemic among MSM. Participants were also assigned to one of three monthly income groups (0–4849 RM; 4850–10,959 RM; 10,960+ RM; corresponding to 0–1071 USD; 1071–2420 USD; 2420+ USD), representing the bottom 40%, middle 40%, and top 20% of income earners in Malaysia according to the 2019 Household Income & Basic Amenities Survey Report by the Department of Statistics [24].

### 3.2. Psychosocial Factors

Participants were asked about their experience of depressive symptoms in the past two weeks using the PHQ-2 scale, a two-item screening scale for depression. PHQ-2 scores range from 0–6, with a score of 3 or higher indicating likely major depression disorder [25].

We also asked participants two questions about stigma and acceptance of their sexual orientation: “Have you told your family about your sexual orientation?” (*‘No’; ‘Yes, but only a few family members know’; ‘Yes, most of my family members know of my sexual orientation /identity’*); and “Has a healthcare provider ever discriminated against you because of your sexual orientation? For example, treating you unfairly or denying you care/treatment”. Participants were re-coded as ‘out’ to their family if they chose an answer other than ‘No’.

### 3.3. PrEP Knowledge and History

Participants were asked whether they had ever heard of PrEP before this survey and if they had ever used PrEP. If participants indicated having used PrEP, they were then asked if they were currently using PrEP. Participants were also asked if they knew anyone using PrEP, if they were willing to use (or continue using) PrEP to reduce their risk of contracting HIV, and if they had ever shared their PrEP pills with another person.

### 3.4. Recent Sexual and HIV-Related Behavior

Participants were asked for information about their sexual behavior in the previous 6 months, including: engagement in anal sex with another man, having an HIV-positive sexual partner, consistent condom use during anal sex (defined as having always used a condom during anal sex), having been paid to have sex; and receiving a diagnosis for a sexually transmitted infection (STI) other than HIV (e.g., Chlamydia, Gonorrhea, Syphilis). We also asked participants if they had taken an HIV test and injected drugs in the previous 6 months.

Participants were asked to self-assess their perceived risk of contracting HIV in the following 6 months using a four-point rating scale (‘not likely at all’; ‘a little likely’; ‘somewhat likely’; ‘extremely likely’).

## 4. Data Analysis

Data analyses were performed using IBM SPSS v. 22 (IBM Corporation, Armonk, NY, USA) [26]. We computed descriptive statistics, including frequencies and percentages for categorical variables. We performed chi-square tests to identify differences in demographic characteristics, psychosocial factors, PrEP knowledge and history, and recent behaviors between participants who had engaged in chemsex in the previous 6 months and participants who had not. Independent variables associated with recent engagement in chemsex at *p* < 0.10 by the chi-square tests were included in a multivariable logistic regression analysis of correlates of recent chemsex engagement. Covariates in the multivariate logistic regression analysis were evaluated for statistical significance at the 95% confidence intervals. Non-significant covariates were progressively eliminated from the model through backward elimination.

## 5. Results

### 5.1. Participant Characteristics

Participant characteristics are described in Table 1. Most participants were 18–29 (43.2%) or 30–39 (39.2%) years old. Most participants identified as Chinese (49.2%) or Malay (29.3%) ethnicities. Over half of the participants were university graduates (65.9%) and single (70.7%). The majority of participants (63.2%) were in the bottom 40% of income earners, with a monthly income below RM 4849. More than two-thirds of participants (69.1%) had not disclosed their sexual orientation to anyone in their family and 12.9% reported having experienced discrimination by a healthcare provider because of their sexual orientation.

Overall, 9.0% of participants reported to have engaged in chemsex in the past 6 months. Almost two-thirds (66.2%) of participants reported having tested for HIV in their life, and of those *(n* = 576), half (290/576; 50.3%) reported that they had tested for HIV in the past 6 months. In terms of sexual behaviors, over half of participants reported having had anal sex in the past 6 months (62.3%), and of those (*n* = 542), more than half (330/542; 60.9%) reported engaging in condomless anal sex. A minority of participants reported having an HIV-positive sexual partner (9.9%), having been paid for sex (5.6%), and injected drugs (3.1%).

In this sample, 17.8% of participants reported having used PrEP before. Of those (*n* = 155), more than half (96/155; 61.9%) were currently using PrEP. Almost all participants were willing to use PrEP (90.6%), even though a majority believed that they were not likely at all (61.8%) or a little likely (25.6%) to acquire HIV in the next 6 months.

### 5.2. Correlates of Chemsex Engagement

Table 1 shows the bivariate associations between demographic characteristics, psychosocial factors, PrEP knowledge and history, recent sexual and HIV-related behavior, and recent engagement in chemsex. Table 2 shows independent correlates of recent chemsex engagement in the full multivariate logistic regression model. Table 3 shows the final multivariable logistic regression model after backward elimination. Knowing someone using PrEP (aOR = 2.93; 95% CI, 1.62–5.31), having shared PrEP with someone else (aOR = 5.60; 95% CI, 1.76–17.77), higher perceived HIV risk (aOR = 3.15; 95% CI, 1.25–7.93), recent injection drug use (aOR = 6.61; 95% CI, 2.30–19.03), recent engagement in anal sex (aOR = 2.21; 95% CI, 1.07–4.57), recent sexual intercourse with an HIV-positive partner (aOR = 2.09; 95% CI, 1.07–4.08), recent engagement in transactional sex (aOR = 2.38; 95% CI, 1.06–5.39), and a recent diagnosis of an STI other than HIV (aOR = 2.36; 95% CI, 1.25–4.44) were positively associated with recent engagement in chemsex. 

### 5.3. Discussion

Chemsex practices are scantly documented in Malaysia, where drug use and same-sex sexual behavior are highly stigmatized, discriminated against, and criminalized. To our knowledge, this is the first study to assess engagement in chemsex and associated factors among Malaysian MSM. This study provides important insights into the chemsex practice and implications for future interventions among this highly marginalized group. Overall, one in eleven (9.0%) MSM reported having recently engaged in chemsex. Although slightly lower than observed in previous studies among Malaysian MSM [27,28], this prevalence falls within the range observed among MSM elsewhere in Asia [19,28,29]. In line with previous studies [19], the findings indicate that chemsex users are a heterogeneous group, as chemsex was comparably practiced by MSM of various sociodemographic characteristics (including age, ethnicity, educational level, and income), rather than being concentrated in specific communities. 

Participants in our sample also faced several vulnerabilities to HIV, including condomless anal sex and engagement in transactional sex, all of which have been central to the increasing HIV epidemic in the country [22,30,31,32]. Worsening the concern of sexual risk is the increased odds of injection drug use, sexual relationships with HIV-positive partners, increased STI diagnoses, and transactional sex among MSM who engage in chemsex, which is in line with previous studies [5]. For example, a 2021 scoping review of SDU in Asia found that SDU was common among male sex workers (MSW) in the region [10]. Although the studies cited were from other Southeast Asian countries (e.g., Vietnam, the Philippines, Singapore), our findings suggest that the association between transactional sex and chemsex is also common in the Malaysian context. The finding that chemsex participants in our sample were more likely to have had a recent HIV-positive sexual partner is also in line with previous studies, possibly as a coping mechanism for the stigma associated with an HIV diagnosis. Specifically, MSM often attempt to use chemsex to escape emotional pain and feelings of lost social worth [10,19,33]. Experience of discrimination in healthcare settings was higher among chemsex-involved participants compared to those who had not recently engaged in chemsex, although the association was not significant in the multivariable model. Overall, these findings indicate the need for prevention strategies and health policies to be tailored to the particular subgroup of MSM engaged in chemsex to address the specific health vulnerabilities and adverse consequences of this practice. For example, digital health technologies tailored for substance-using MSM could effectively overcome some of the issues of traditional healthcare provision for this minority population by connecting them with LGBT-friendly providers and clinics via telehealth or referrals [34].

Interestingly, those who knew someone using PrEP and had shared PrEP with others were more likely to engage in chemsex, although the current use of PrEP was not associated with chemsex engagement. This suggests that MSM practicing chemsex may share PrEP with other chemsex participants as a harm reduction strategy and are likely to find themselves in distinct social and sexual networks. A peer-to-peer strategy could be employed to engage MSM who participate in chemsex in HIV prevention. Peer-led approaches such as peer outreach workers and peer navigators may, therefore, be a valuable strategy to engage stigmatized populations such as MSM, and may be particularly promising in the context of distinct subcultures such as chemsex [35]. 

Our study has a few limitations. Due to the recruitment of participants through online social networks, the study is likely to have suffered from some level of selection bias. In fact, our sample was not representative of the demographics of the Malaysian population, with an overrepresentation of university graduates and ethnic Chinese and an underrepresentation of ethnic Malays [36]. This could be due to the online format of the survey, which may have led to an overrepresentation of university graduates with higher internet and technology literacy, as well as the fact that the survey was in English, which ethnically Chinese residents of Malaysia tend to be more proficient in [37]. Given that the survey inquired about practices in the previous 6 months, recall bias might also have affected responses. Additionally, the COVID-19 pandemic may have affected the results of our study, as the movement control order imposed in Malaysia (from March 2020 to November 2021) corresponds with the study period. Finally, we cannot claim causality for any of the observed associations. While we examined factors associated with chemsex engagement, we did not explore participants’ practices during chemsex events (e.g., motivations for engagement, venue or environment, drug of choice, sexual partners or behaviors, etc.). For example, while we found recent engagement in transactional sex and chemsex to be associated, we cannot know whether transactional sex occurred in the context of chemsex or independently. Recognizing and understanding the diverse range of motivations and practices associated with chemsex is central to helping MSM manage risk and reduce negative impacts on their health and others’.

## 6. Conclusions

This study constitutes one of the first efforts toward determining the prevalence and factors associated with chemsex among MSM in Malaysia, a group that already faces substantial societal marginalization. While the engagement in chemsex was relatively low among MSM in our sample, the results indicate that MSM practicing chemsex are a heterogeneous group encompassing a broad range of sociodemographic characteristics. HIV/STI risk factors, such as transactional sex, HIV-positive sexual partners, and injection drug use, were independently associated with chemsex, indicating a need for HIV prevention among MSM practicing chemsex. Depressive symptoms were highly prevalent across the entire sample, regardless of chemsex engagement. These findings offer important insights into chemsex practices and the syndemics of psychosocial problems and HIV risk and demonstrate the crucial need for funding, designing, and implementing sexual health interventions for Malaysian MSM to recognize their unique chemsex harm reduction needs. As such, there is an urgent need for broader programs that integrate drug, sexual health, and mental health services, with a focus on harm reduction (e.g., condoms, access to and utilization of HIV testing and PrEP services, drug knowledge, and safer drug use) aimed at MSM who practice chemsex.

## Figures and Tables

**Table 1:** Characteristics of participants stratified by recent engagement in chemsex (*n* = 870).

Variables	Full Sample	Engagement in Chemsex		*p*-value ^d^
	(*n* = 870)	No (*n* = 792)	Yes (*n* = 78)	
Demographic Characteristics	*n* (%)	*n* (%)	*n* (%)	
Age				0.059
18–29	377 (43.3)	353 (44.6)	24 (30.8)	
30–39	341 (39.2)	300 (37.9)	41 (52.6)	
40–49	105 (12.1)	95 (12.0)	10 (12.8)	
50+	47 (5.4)	44 (5.6)	3 (3.8)	
Ethnicity				0.184
Chinese	428 (49.2)	396 (50.0)	32 (41.0)	
Indian	53 (6.1)	47 (5.9)	6 (7.7)	
Malay	255 (29.3)	233 (29.4)	22 (28.2)	
Other	134 (15.4)	116 (14.6)	18 (23.1)	
University graduate ^a^				0.399
No	297 (34.1)	267 (33.7)	30 (38.5)	
Yes	573 (65.9)	525 (66.3)	48 (61.5)	
Relationship status				0.627
Single	615 (70.7)	558 (70.5)	57 (73.1)	
Partner	255 (29.3)	234 (29.5)	21 (26.9)	
Monthly Income				0.172
RM 0–4849	550 (63.2)	508 (64.1)	42 (53.8)	
RM 4850–10,959	257 (29.5)	227 (28.7)	30 (38.5)	
RM 10,960+	62 (7.2)	57 (7.2)	6 (7.7)	
Psychosocial Factors	*n* (%)	*n* (%)	*n* (%)	*p*-value ^d^
Depressive symptoms				0.888
No	564 (64.8)	514 (64.9)	50 (64.1)	
Yes	306 (35.2)	278 (35.1)	28 (35.9)	
Disclosed sexual orientation to family				<0.001
No	601 (69.1)	562 (71.0)	39 (50.0)	
Yes	269 (30.9)	230 (29.0)	39 (50.0)	
Experienced discrimination by healthcare provider				<0.001
No	758 (87.1)	702 (88.6)	56 (71.8)	
Yes	112 (12.9)	90 (11.4)	22 (28.2)	
PrEP ^b^ Knowledge and History	*n* (%)	*n* (%)	*n* (%)	*p*-value
Ever heard of PrEP ^b^				0.140
No	166 (19.1)	156 (19.7)	10 (12.8)	
Yes	704 (80.9)	636 (80.3)	68 (87.2)	
Ever used PrEP ^b^				<0.001
No	715 (82.2)	666 (84.1)	49 (62.8)	
Yes	155 (17.8)	126 (15.9)	29 (37.2)	
Currently using PrEP ^b^	(*n* = 155)	*(n* = 126)	(*n* = 29)	0.660
No	59 (38.1)	49 (38.9)	10 (34.5)	
Yes	96 (61.9)	77 (61.1)	19 (65.5)	
Knows anyone using PrEP ^b^				<0.001
No	449 (51.6)	439 (54.3)	19 (24.4)	
Yes	421 (48.4)	362 (45.7)	59 (75.6)	
Ever shared PrEP ^b^	(*n* = 155)	(*n* = 126)	(*n* = 29)	0.001
No	138 (89.0)	117 (92.9)	21 (72.4)	
Yes	17 (11.0)	9 (7.1)	8 (27.6)	
Willing to use PrEP ^b^				0.339
No	82 (9.4)	77 (9.7)	5 (6.5)	
Yes	788 (90.6)	715 (90.3)	73 (93.6)	
Likelihood of contracting HIV in next 6 months				<0.001
Not likely at all	538 (61.8)	506 (63.9)	32 (41.0)	
A little likely	223 (25.6)	199 (25.1)	24 (30.8)	
Somewhat likely	68 (7.8)	58 (7.3)	10 (12.8)	
Extremely likely	41 (4.7)	29 (3.7)	12 (15.4)	
Lifetime HIV testing	*n* (%)	*n* (%)	*n* (%)	*p*-value
Ever tested for HIV				0.022
No	294 (33.8)	276 (34.8)	18 (23.1)	
Yes	576 (66.2)	516 (65.2)	60 (76.9)	
Recent Behavior (Past 6 months)	*n* (%)	*n* (%)	*n* (%)	*p*-value
HIV test	(*n* = 576)	(*n* = 516)	(*n* = 60)	0.446
No	286 (49.7)	259 (50.2)	27 (45.0)	
Yes	290 (50.3)	257 (49.8)	33 (55.0)	
Injected drugs				<0.001
No	843 (96.9)	778 (98.2)	65 (83.3)	
Yes	27 (3.1)	14 (1.8)	13 (16.7)	
Engaged in anal sex				<0.001
No	328 (37.7)	317 (40.0)	11 (14.1)	
Yes	542 (62.3)	475 (60.0)	67 (85.9)	
Consistent condom use	(*n* = 542)	(*n* = 475)	(*n* = 67)	0.166
No	330 (60.8)	277 (58.3)	53 (79.1)	
Yes	212 (39.1)	198 (41.7)	14 (20.9)	
HIV-positive sexual partner				<0.001
No	784 (90.1)	728 (91.9)	56 (71.8)	
Yes	86 (9.9)	64 (8.1)	22 (28.2)	
Engaged in transactional sex				<0.001
No	821 (94.4)	758 (95.7)	63 (80.8)	
Yes	49 (5.6)	34 (4.3)	15 (19.2)	
Diagnosed with STI ^c^				<0.001
No	767 (88.2)	717 (90.5)	50 (64.1)	
Yes	103 (11.8)	75 (9.5)	28 (35.9)	

^a^ Includes college, university, professional degree; ^b^ PrEP: pre-exposure prophylaxis; ^c^ STI: sexually transmitted infections (includes gonorrhea, chlamydia, syphilis, hepatitis B, hepatitis C); ^d^ *p*-value for χ^2^ test.

**Table 2:** Full multivariate logistic regression analysis of correlates of recent engagement in chemsex.

Variable	*B*	SE	*p*	aOR	95% CI
Demographic Characteristics					
Age					
18–29	-	-	-	-	-
30–39	0.38	0.31	0.219	1.46	[0.80, 2.67]
40–49	0.34	0.45	0.458	1.40	[0.56, 3.41]
50+	−0.09	0.72	0.906	0.92	[0.22, 3.79]
Psychosocial Factors					
Disclosed sexual orientation to family	0.43	0.28	0.123	1.53	[0.89, 2.64]
Experienced discrimination by healthcare provider	0.62	0.34	0.071	1.86	[0.95, 3.63]
Lifetime HIV testing					
Ever tested for HIV	−0.22	0.33	0.504	0.80	[0.42, 1.53]
PrEP ^a^ Knowledge and History					
Ever used PrEP ^a^	0.04	0.34	0.913	1.04	[0.53, 2.02]
Knows someone using PrEP ^a^	1.05	0.32	0.001	2.87	[1.54, 5.33]
Ever shared PrEP ^a^	1.42	0.65	0.028	4.14	[1.16, 14.75]
Likelihood of contracting HIV in next 6 months					
Not likely at all (reference)	-	-	-	-	-
A little likely	0.46	0.32	0.150	1.58	[0.85, 2.96]
Somewhat likely	0.20	0.49	0.690	1.22	[0.47, 3.18]
Extremely likely	1.07	0.48	0.028	2.90	[1.13, 7.48]
Recent Behavior (Past 6 months)					
Injected drugs	1.77	0.56	0.001	5.88	[1.99, 17.47]
Engaged in anal sex	0.72	0.38	0.054	2.06	[0.99, 4.30]
HIV-positive sexual partner	0.70	0.36	0.049	2.02	[1.00, 4.07]
Engaged in transactional sex	0.89	0.42	0.036	2.44	[1.06, 5.60]
Diagnosed with STI ^b^	0.83	0.34	0.014	2.29	[1.18, 4.43]

^a^ PrEP: pre-exposure prophylaxis; ^b^ STI: sexually transmitted infections (includes gonorrhea, chlamydia, syphilis, hepatitis *B*, hepatitis C).

**Table 3:** Final multivariate logistic regression analysis of correlates of recent engagement in chemsex after backward elimination.

Variable	*B*	SE	*p*	aOR	95% CI
PrEP ^a^ Knowledge and History					
Knows someone using PrEP ^a^	1.05	0.32	<0.001	2.93	[1.62, 5.31]
Ever shared PrEP ^a^	1.42	0.65	0.003	5.60	[1.76, 17.77]
Likelihood of contracting HIV in next 6 months					
Not likely at all	-	-	-	-	-
A little likely	0.46	0.32	0.146	1.58	[0.85, 2.92]
Somewhat likely	0.20	0.49	0.652	1.31	[0.53, 3.23]
Extremely likely	1.07	0.48	0.015	3.15	[1.25, 7.93]
Recent Behavior (Past 6 months)					
Injected drugs	1.77	0.56	<0.001	6.61	[2.30, 19.03]
Engaged in anal sex	0.72	0.38	0.032	2.21	[1.07, 4.57]
HIV-positive sexual partner	0.70	0.36	0.032	2.09	[1.07, 4.08]
Engaged in transactional sex	0.89	0.42	0.037	2.38	[1.06, 5.39]
Diagnosed with STI ^b^	0.83	0.34	0.008	2.36	[1.25, 4.44]

^a^ PrEP: pre-exposure prophylaxis; ^b^ STI: sexually transmitted infections (includes gonorrhea, chlamydia, syphilis, hepatitis *B*, hepatitis C).

## Data Availability

The data that support the findings of this study are available from the authors upon reasonable request.
